# Inhibition of nicotinic acetylcholine receptors by oligoarginine peptides and polyamine-related compounds

**DOI:** 10.3389/fphar.2023.1327603

**Published:** 2023-12-15

**Authors:** Lucy O. Ojomoko, Elena V. Kryukova, Natalya S. Egorova, Arthur I. Salikhov, Lyubov A. Epifanova, Daria A. Denisova, Alex R. Khomutov, Dmitry A. Sukhov, Alexander A. Vassilevski, Maxim A. Khomutov, Victor I. Tsetlin, Irina V. Shelukhina

**Affiliations:** ^1^ Shemyakin-Ovchinnikov Institute of Bioorganic Chemistry, Russian Academy of Sciences, Moscow, Russia; ^2^ Engelhardt Institute of Molecular Biology, Russian Academy of Sciences, Moscow, Russia; ^3^ Moscow Institute of Physics and Technology (State University), Moscow, Russia

**Keywords:** nicotinic acetylcholine receptor, oligoarginine, polyamines, acylpolyamine, radioligand analysis, electrophysiology, calcium imaging, argiopin

## Abstract

Oligoarginine peptides, known mostly for their cell-penetrating properties, are also inhibitors of the nicotinic acetylcholine receptors (nAChRs). Since octa-arginine (R8) inhibits α9α10 nAChR and suppresses neuropathic pain, we checked if other polycationic compounds containing amino and/or guanidino groups could be effective and tested the activity of the disulfide-fixed “cyclo”R8, a series of biogenic polyamines (putrescine, spermidine, and spermine), *C*-methylated spermine analogs, agmatine and its analogs, as well as acylpolyamine argiotoxin-636 from spider venom. Their inhibitory potency on muscle-type, α7 and α9α10 nAChRs was determined using radioligand analysis, electrophysiology, and calcium imaging. “Cyclo”R8 showed similar activity to that of R8 against α9α10 nAChR (IC_50_ ≈ 60 nM). Biogenic polyamines as well as agmatine and its analogs displayed low activity on muscle-type *Torpedo californica*, as well as α7 and α9α10 nAChRs, which increased with chain length, the most active being spermine and its *C*-methylated derivatives having IC_50_ of about 30 μM against muscle-type *T. californica* nAChR. Argiotoxin-636, which contains a polyamine backbone and terminal guanidino group, also weakly inhibited *T. californica* nAChR (IC_50_ ≈ 15 μM), but it revealed high potency against rat α9α10 nAChR (IC_50_ ≈ 200 nM). We conclude that oligoarginines and similar polycationic compounds effectively inhibiting α9α10 nAChR may serve as a basis for the development of analgesics to reduce neuropathic pain.

## 1 Introduction

Oligoarginine peptides as such or in polymeric vesicles are widely used for intracellular delivery of a wide variety of compounds [see reviews (Ruan et al., 2016; Xie et al., 2021)]. Positively charged oligo- and polyarginines exhibit numerous biological activities (Edwards et al., 2020). However, the molecular mechanisms of their action are poorly understood and there is almost no information on their binding to specific molecular targets. An exception is the interaction of oligoarginines containing from 8 to 18 arginine residues (R8 to R18) with *N*-methyl-D-aspartate (NMDA)-type glutamate receptors (MacDougall et al., 2017). These oligoarginines are considered as a new class of neuroprotective molecules for the treatment of a range of neurological disorders (Meloni et al., 2020).

It was found that oligoarginines containing from 6 to 16 arginine residues (R6 to R16) interact with both muscle and neuronal nicotinic acetylcholine receptors (nAChRs), while the potency of inhibition of a particular receptor subtype depends on the peptide length (Lebedev et al., 2019). Binding to nAChRs was studied not only for oligoarginines, but also for oligohistidines and oligolysines, and it was shown that oligoarginines of sufficient length are the most effective (Zhang et al., 2022).

**FIGURE 1:**
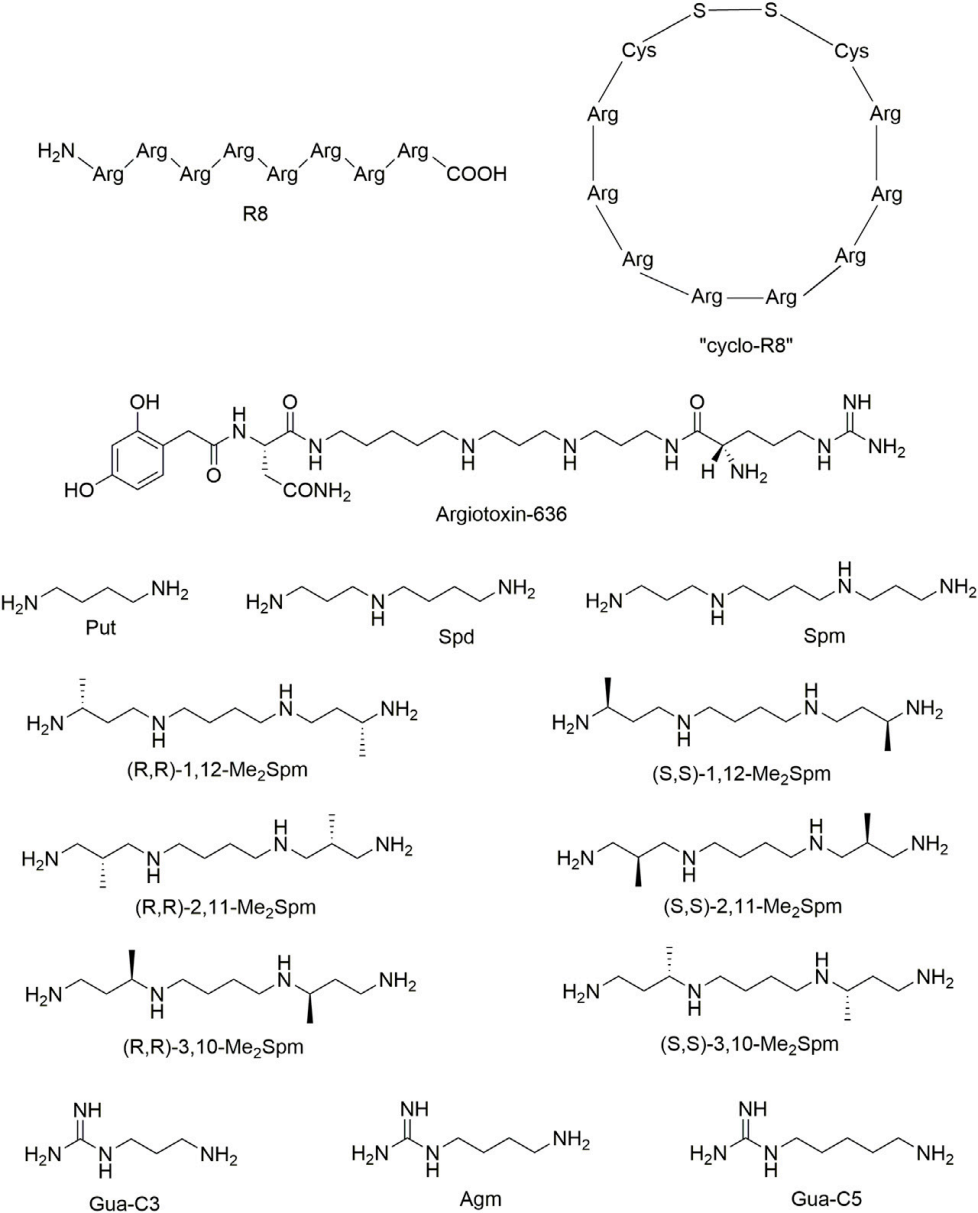
The chemical structures of tested polycationic compounds: octa-arginine R8 and “cyclo”R8, argiotoxin-636, putrescine (Put), spermidine (Spd), spermine (Spm), *C*-methylated spermine analogs (Me_2_Spm), agmatine (Agm) and its derivatives guanidine-C3 (Gua-C3) and guanidine-C5 (Gua-C5). The structures were drawn using ChemDraw Professional 18.0.

It was established that octa-arginine R8 (Figure 1), which has a high affinity for α9α10 nAChR, reduces neuropathic pain with the same efficacy as α-conotoxin RgIA, a specific inhibitor of this receptor subtype (Dyachenko et al., 2022). In this regard, we decided to obtain a more metabolically stable derivative of R8. Cyclization is a standard method to increase the proteolytic resistance of pharmacologically active peptides, because the lack of free amino and carboxyl groups of *N-* and *C*-terminal amino acids makes cyclic peptides resistant towards blood exopeptidases (Costa et al., 2023). The structures of the oligoarginine peptides can be stabilized by various modes of cyclization to improve their stability and cell-penetrating properties (Bató et al., 2023). For example, on the basis of R8 its cyclic fluorescent analog has been prepared (Fang et al., 2022). Here, we stabilize the R8 conformation by attaching cysteine residues at its *N-* and *C*-termini and closing the disulfide bond between them (“cyclo”R8, Figure 1). We have recently used the same approach for the synthesis of the fragment of a three-finger protein, which preserved the structure of the central loop and displayed the activity of the parent protein in binding to muscle-type nAChR (Kryukova et al., 2019; Mineev et al., 2020). For “cyclo”R8, we compared its inhibitory potency with that of the linear R8 on the muscle-type, neuronal α7 and on α9α10 nAChRs normaly present on the cochlear cells and in the immune system [see review (Elgoyhen, 2023)].

Since a number of positively charged oligopeptides inhibit nAChRs (Zhang et al., 2022), here we analyzed other low-molecular-mass polycationic compounds containing amino and guanidino groups. We started with biogenic polyamines putrescine (Put), spermidine (Spd), spermine (Spm), *C*-methylated Spm analogs, and agmatine (Agm) and its analogs (Figure 1). Being polycations under physiological pH, biogenic polyamines effectively interact with different negatively charged cellular targets, including nucleic acids, receptors and other proteins (Matta et al., 2021). Polyamines play an important role in synaptic transmission by conferring inward rectification to certain potassium channels, α-amino-3-hydroxy-5-methyl-4-isoxazolepropionate (AMPA)-type glutamate receptors and nAChRs (Haghighi and Cooper, 2000; Nichols and Lee, 2018; Twomey et al., 2018). Recently, it has been shown that polyamines selectively control the biogenesis of α7 or α4β2 nAChR receptors (Dhara et al., 2020). Analogs and derivatives of polyamines are widely used to investigate cellular functions of Spm and Spd (Holbert et al., 2022), while *C*-methylated analogs of polyamines are one of the research tools providing functionally active mimetics of Spm and Spd (for reviews see (Keinänen et al., 2014; Khomutov et al., 2019a)). Polyamines are accumulated in glial cells (astrocytes, oligodendrocytes, Müller and Bergmann glia), but not in neurons and this makes polyamine-reservoirs in brain/retina restricted to glial cells (Rieck et al., 2022; Ríos et al., 2023; Weiss et al., 2023). These cells express a variety of polyamine specific and nonselective organic cation transporters such as SLC22A-1,2,3 (Sala-Rabanal et al., 2013), SLC18B1 (Hiasa et al., 2014), and ATP13A2 (Qiao et al., 2016; Vrijsen et al., 2023) for polyamine uptake into the cytoplasm from any extracellular sources such as cerebral spinal fluid (Weiss et al., 2023). Since glial cells may take up di- and polyamine analogs and derivatives (Malpica-Nieves et al., 2021), these cells may also take up *C*-methylated analogs of Spm and Agm of this study.

Another polycationic compound of our choice is argiotoxin-636 (AgTx-636), also known as argiopin, purified from *Argiope lobata* spider venom. It is the first-in-class molecule belonging to the so-called acylpolyamines; AgTx-636 has a polyamine tail linked to an aromatic acyl head group and a terminal arginine residue (Figure 1) (Grishin et al., 1986; Antonov et al., 1987; Grishin et al., 1989). Binding of AgTx-636 to both AMPA and NMDA-type glutamate receptors was studied in detail (Davies et al., 1992; Twomey et al., 2018). In addition, it is known to inhibit weakly some neuronal nAChRs (Liu et al., 1997), but there are no data on its interaction with α9α10 nAChR. For a number of derivatives of another well-studied acylpolyamine philanthotoxin-433 (PTX-433), highly potent inhibition of α3β4 and α4β2 nAChRs was demonstrated (Kachel et al., 2019), but the action on α9α10 nAChR was not tested either.

In this study we tested the inhibitory potency of a number of polycationic compounds on α9α10 nAChR, a known molecular target to reduce neuropathic pain (Hone and McIntosh, 2023; Shelukhina et al., 2023), as well as on muscle and α7 nAChRs to check the possibility of undesirable side effects. Muscle relaxant activity mediated by controlled muscle nAChR inhibition (Shelukhina et al., 2018) may be profitable in complex analgesic therapy, but excessive affinity to muscle nAChR may lead to paralysis as in the case of the selective snake α-neurotoxins or α-conotoxins. Potentiation of α7 nAChR is beneficial in chronic pain, mostly by alleviating neuroinflammation (Zhou et al., 2022; Shelukhina et al., 2023), therefore, it is necessary to control its inhibition, which may lead to the opposite effect *in vivo*.

## 2 Methods

### 2.1 Synthesis of linear octa-arginine R8 and disulfide-stabilized “cyclo” R8

The peptides were prepared by solid-phase synthesis using Fmoc/t-butyl strategy on tritylchloride-polystyrene resin (Intavis, Tubingen, Germany). The disulfides were formed under conventional oxidation conditions: prolonged incubation on air in 50% aqueous acetonitrile at room temperature in the presence of N-ethyldiisopropylamine (pH 8.0). The peptides purity (>98%) was confirmed using reversed-phase high-performance liquid chromatography (RP-HPLC) and the respective monoisotopic molecular masses (MW (R8) = 1266.82 Da, MW (“cyclo”R8) = 1470.84 Da) were determined by matrix-assisted laser desorption/ionization mass-spectrometry (MALDI-MS) using Ultraflex I mass-spectrometer (Bruker Daltonik, Bremen, Germany). Peptide aliquots were prepared immediately after lyophylization by dissolving the dried compound in water of Milli-Q grade; the aliquots were stored at –20°C. Peptide concentration was determined by dissolving a weighted amount in a fixed volume of solution.

### 2.2 Synthesis of bis-methylated spermine analogs

(R,R)-2,13-Diamino-5,10-diazatetradecane and (S,S)-2,13-diamino-5,10-diazatetradecane tetrahydrochlorides, (R,R)-1,12-Me_2_Spm and (S,S)-1,12-Me_2_Spm were synthesized as described (Grigorenko et al., 2007).

(R,R)-1,12-Diamino-3,10-dimethyl-4,9-diazadodecane and (S,S)-1,12-diamino-3,10-dimethyl-4,9-diazadodecane tetrahydrochlorides, (R,R)-3,10-Me_2_Spm and (S,S)-3,10-Me_2_Spm were synthesized as described (Khomutov et al., 2020).

(R,R)-1,12-diamino-2,11-dimethyl-4,9-diazadodecane and (S,S)-1,12-diamino-2,11-dimethyl-4,9-diazadodecane tetrahydrochlorides, (R,R)-2,11-Me_2_Spm and (S,S)-2,11-Me_2_Spm were synthesized from (R)- and (S)-3-amino-2-methylpropanol-1 as described (Khomutov et al., 2019b).

### 2.3 AgTx-636 purification

AgTx-636 was isolated and purified from *A. lobata* venom as described (Grishin et al., 1989; Twomey et al., 2018). Briefly, the lyophilized venom was dissolved in water, mixed with ethanol, centrifuged, and the supernatant was separated by RP-HPLC. AgTx-636 was purified to homogeneity (>98%). AgTx-636 aliquots were freeze-dried; one was dissolved in MeCN–MeOH, and the concentration was determined by UV absorbance using the known extinction coefficient (ε_280_ = 4150). Other aliquots were dissolved in water or buffer solution to the desired concentrations.

### 2.4 Ca-imaging analysis of inhibition of α7 nAChR endogenously expressed in neuroblastoma SH-SY5Y cells

Human neuroblastoma SH-SY5Y cells were cultured in DMEM/F12 medium (Thermo Fisher Scientific, Waltham, MA, United States) supplemented with 10% fetal bovine serum (FBS) (PAA Laboratories, Pasching, Austria) in a CO_2_ incubator at 37°C and 5% CO_2_ atmosphere. After removing the growth medium, SH-SY5Y cells were loaded with Fluo-4 Direct Calcium Assay Kit (Thermo Fisher Scientific) for 30 min at 37°C and then were kept for 30 min at room temperature according to the manufacturer’s protocol. Then SH-SY5Y cells were preincubated with positive allosteric modulator of α7 nAChR, PNU120596 (10 μM, Tocris Bioscience, Bristol, United Kingdom), and inhibitors (R8 and “cyclo”R8, Agm, Spd, Spm, *rac*-2,11-Me_2_Spm, AgTx-636 and α-cobratoxin (CTX)) in a buffer containing 140 mM NaCl, 2 mM CaCl_2_, 2.8 mM KCl, 4 mM MgCl_2_, 20 mM HEPES, 10 mM glucose, pH 7.4 for 20 min before agonist PNU282987 (200 nM, Tocris Bioscience) addition. The Fluo-4 fluorescence was analyzed with the microplate reader Hidex Sence (Hidex, Turku, Finland) (excitaion/emission 485/535 nm). Data files were analyzed using Hidex Sence software and OriginPro 2015 software (OriginLab, Northampton, MA, United States).

### 2.5 Competition radioligand assay with ^125^I-labeled α-bungarotoxin

The binding capacity (IC_50_) of the low-molecular-mass compounds was evaluated by competition with ^125^I-labeled α-bungarotoxin (^125^I-αBgt) as described (Kryukova et al., 2019; Mineev et al., 2020). The suspensions of membranes from *Torpedo californica* ray electric organ or human α7 nAChR expressed in GH4C1 cells (1.25 nM or 0.4 nM αBgt binding sites, respectively) were incubated in 50 μL of binding buffer (20 mM Tris-HCl, pH 8.0, containing 1 mg/mL bovine serum albumin) for 90 min with various amounts of low-molecular-mass compounds. Then, 0.1–0.2 nM ^125^I-αBgt (500 Ci/mmol) was added and incubated for another 5 min. The *T. californica* membranes or cell suspensions were applied to glass GF/C filters (Whatman, Maidstone, United Kingdom) presoaked in 0.3% polyethylenimine. Filter washing, measurement of bound radioactivity and non-specific binding of ^125^I-αBgt were performed as in (Kryukova et al., 2019; Mineev et al., 2020).

### 2.6 Two-electrode voltage clamp analysis of rat and human α9α10 nAChR inhibition by polycationic compounds

*Xenopus laevis* frogs were fed twice a week and maintained according to supplier recommendations (https://www.enasco.com/page/xen_care). All experiments were carried out in strict accordance with the World Health Organization’s International Guiding Principles for Biomedical Research Involving Animals. The protocol (number 251/2018 26.02.18) was approved by the Institutional Animal Care and Use Committee based on the Institutional Policy on the Use of Laboratory Animals of the Shemyakin-Ovchinnikov Institute of Bioorganic Chemistry Russian Academy of Sciences.

Oocytes were removed from mature anesthetized *X*. *laevis* frogs by dissecting the abdomen and removing necessary amounts of ovarium. Stage V–VI *X*. *laevis* oocytes were defolliculated with collagenase type I (2 mg/mL, Life Technologies, Camarillo, CA, United States) at room temperature (21°C–24°C) for 2 h in Ca^2+^-free Barth’s solution composed of (in mM) 88 NaCl, 1.1 KCl, 2.4 NaHCO_3_, 0.8 MgSO_4_ and 15 HEPES, pH 7.6. Oocytes were injected with 9.2 ng of rat or human nAChR α9 and α10 cRNA (at a ratio of 1:1). Oocytes were incubated at 18°C for 2–4 days before electrophysiological recordings in Barth’s solution composed of (in mM) 88 NaCl, 1.1 KCl, 2.4 NaHCO_3_, 0.3 Ca(NO_3_)_2_, 0.4 CaCl_2_, 0.8 MgSO_4_ and 15 HEPES at pH 7.6, supplemented with 40 μg/mL gentamicin and 100 μg/mL ampicillin. Recordings were performed using a turbo TEC-03X amplifier (NPI Electronic, Tamm, Germany) and WinWCP recording software (University of Strathclyde, Glasgow, United Kingdom). The glass recording electrodes were filled with 3 M KCl and the electrode resistance was 0.1–0.5 MΩ. Membrane potential was clamped at −60 mV. Oocytes were briefly washed with Ba^2+^ Ringer’s solution composed of (in mM) 115 NaCl, 2.5 KCl, 1.8 BaCl_2_ and 10 HEPES, pH 7.2, followed by three applications of 500 μM acetylcholine (ACh). Washout with Ba^2+^ Ringer’s was performed for 5 min between ACh applications. Oocytes were preincubated with various concentrations of “cyclo”R8, *rac*-2,11-Me_2_Spm, Spm, and AgTx-636 for 1 min followed by their co-application with ACh. The peak current amplitudes of ACh-induced responses were measured before (ACh alone) and after the preincubation of oocytes with the inhibitors. The ratio between these two measurements was used to assess the activity of the tested compounds.

Rat α9 and α10 cDNAs was cloned in the pGEMHE vector; human α9 and α10 cDNAs were derived from the pT7TS vector. Plasmid pT7TS and pGEMHE constructs were linearized with XbaI and NheI (NEB, Ipswich, United States) restriction enzymes, respectively. mRNAs were transcribed *in vitro* using T7 or SP6 mMESSAGE mMACHINE High Yield Capped RNA Transcription Kit (Thermo Fisher Scientific). Transcribed mRNA was polyadenylated using the Poly-A-Tailing Kit (Thermo Fisher Scientific). The mRNAs were stored up to 6 months at −70°C.

### 2.7 Statistical analysis

Calcium imaging, radioligand analysis and electrophysiological data were statistically analyzed using OriginPro 7.5 software (Microcal, Northampton, MA, United States) and SigmaPlot 11.0 (Systat Software Inc., CA, United States). The data were first analyzed for normality using the Shapiro-Wilk test, then they were processed using one-way ANOVA test, Tukey’s test, or Student’s t-test, *p < 0.05.

## 3 Results

### 3.1 Inhibition of α7 and α9α10 nAChRs by “cyclo”R8

**FIGURE 2:**
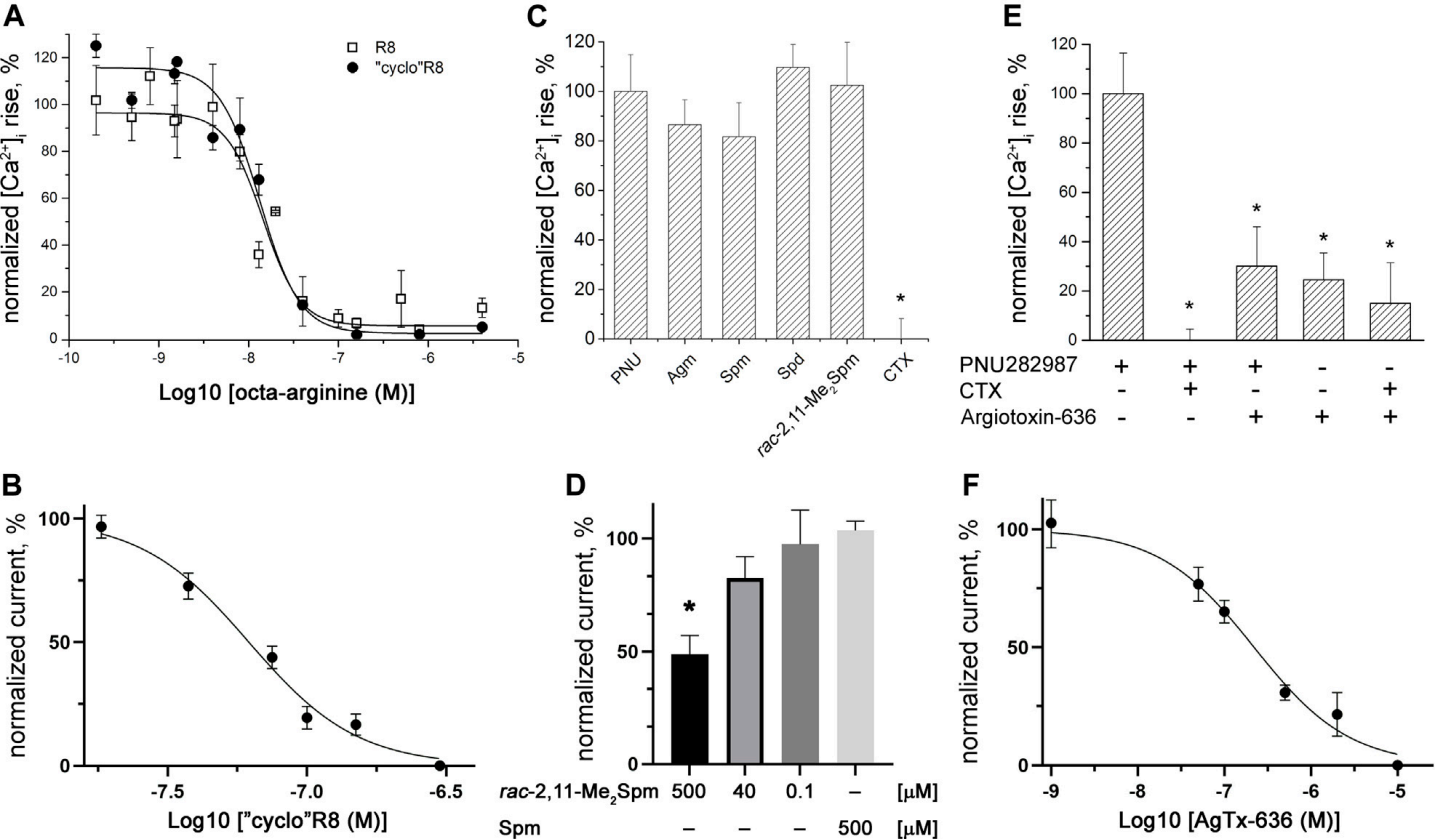
Inhibitory action of polycationic compounds toward α7 (*upper panel*) and α9α10 (*lower panel*) nAChRs. Calcium imaging of **(A)** R8 (IC_50_ = 14.5 ± 1.0 nM) and “cyclo”R8 (IC_50_ = 13.3 ± 3.0 nM), **(C)** 500 μM Agm, Spm, Spd, *rac*-2,11-Me_2_Spm, and 5 μM CTX, and **(E)** 10 μM AgTx-636 and 5 μM CTX inhibition of 200 nM PNU282987-evoked intracellular calcium concentration [Ca^2+^]_i_ rise in SH-SY5Y neuroblastoma cells expressing human α7 nAChR. Electrophysiological recordings of **(B)** “cyclo”R8 (IC_50_ = 61.8 ± 16.0 nM), **(D)** *rac*-2,11-Me_2_Spm and Spm, and **(F)** AgTx-636 inhibition (IC_50_ = 220 ± 22 nM) of ACh (500 μM)-evoked currents in *X*. *laevis* oocytes expressing α9α10 nAChR. Each plot point represents data obtained from three independent experiments [**(A, B, D, F)** mean ± SEM; **(C,E)** mean ± SD, one-way ANOVA test, Tukey’s test, *p < 0.05].

We prepared “cyclo”R8 (Figure 1) by solid-phase peptide synthesis and studied its activity in comparison with the parent R8 using electrophysiology and calcium imaging. We found that “cyclo”R8 showed high affinity to human α7 and α9α10 nAChRs; its inhibitory potency (IC_50_ = 13.3 ± 3.0 nM on α7 and 61.8 ± 16.0 nM on α9α10) was similar to that of R8 (IC_50_ = 14.5 ± 1.0 nM on α7 and 44 (95% CI = 26–75) nM on α9α10 (Lebedev et al., 2019); Figures 2A, B). Thus, the “cyclic” structure of R8 peptide, formed by the addition of two flanking cysteine residues and closing the disulfide bridge between them, did not affect significantly the pharmacological properties toward nAChRs.

### 3.2 Interaction of biogenic polyamines and their analogs with muscle-type, α7 and α9α10 nAChRs

**FIGURE 3:**
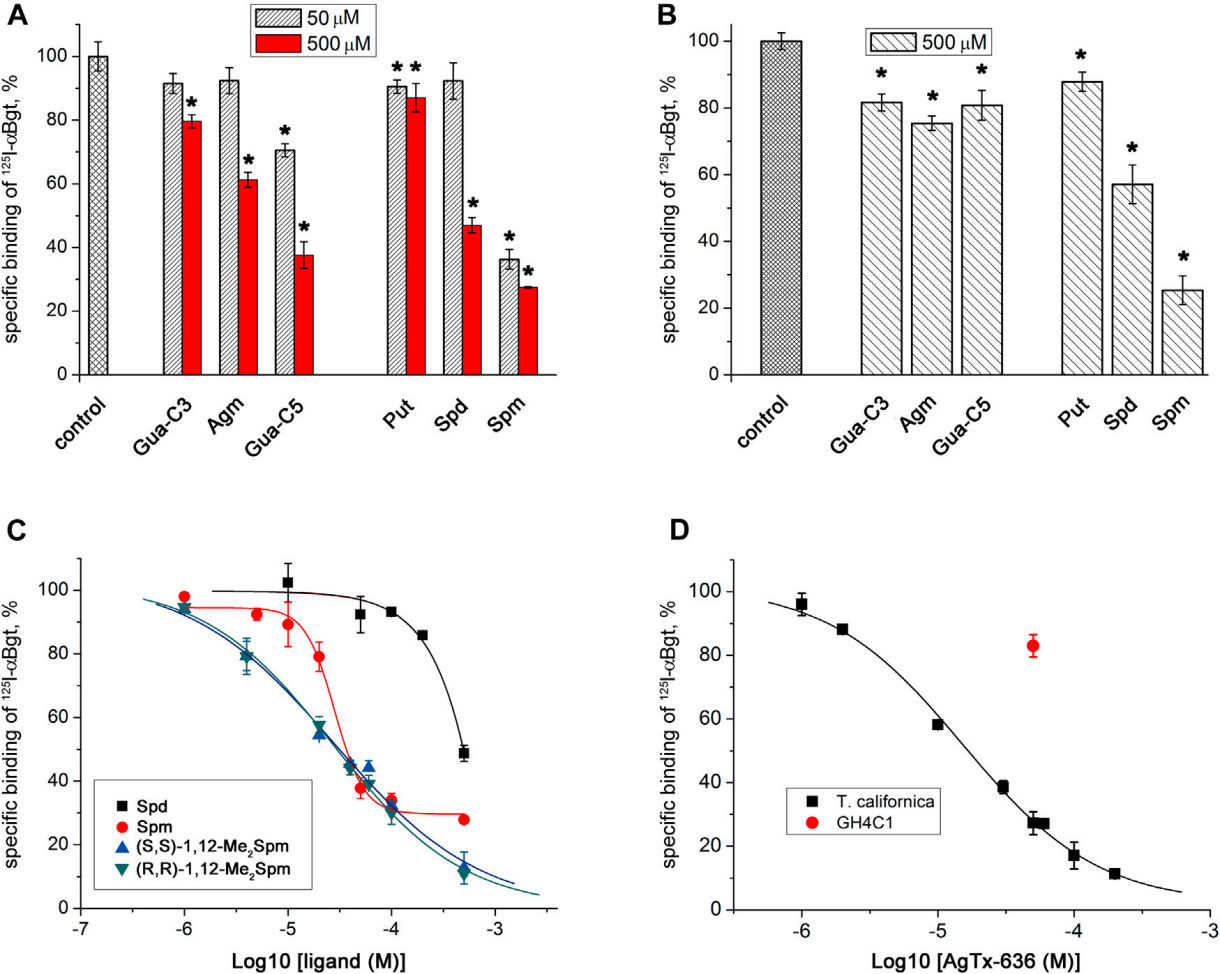
Inhibition of ^125^I-αBgt specific binding to nAChRs by natural polyamines, agmatine and its analogs, diastereomers of *bis*-methylated Spm, and AgTx-636. Bar presentation of the activity of the compounds against **(A)** *T. californica* nAChR and **(B)** human α7 nAChR in GH4C1 cells. **(C)** Dose-response curves of inhibition of ^125^I-αBgt binding to *T. californica* nAChR by Spd, Spm, and two diastereomers of 1,12-Me_2_Spm. **(D)** Radioligand assay of AgTx-636 competition with ^125^I-αBgt for binding to (■) *T. californica* nAChR (IC_50_ = 15.3 ± 1.3 μM) and (●) human α7 nAChR expressed in GH4C1 cells. Specific binding of ^125^I-αBgt in the absence of compounds was accepted as 100%. Each point is represented as the mean ± SEM of 2–4 independent experiments, Student’s t-test, *p < 0.05. IC_50_ values are presented in Table 1.

Competitive radioligand analysis with ^125^I-αBgt was used to test the ability of all selected low-molecular-mass compounds containing amino or guanidino groups (Figure 1), to bind to muscle-type nAChR in *T. californica* membranes and to human neuronal α7 nAChR expressed in GH4C1 cells (Figure 3; Table 1). Binding capacity of Put, Spd, and Spm increased at longer chain of the polyamine. Spm was the most active at concentrations of 500 and 50 μM on both muscle-type *T. californica* nAChR (Figure 3A) and human neuronal α7 nAChR (Figure 3B). Binding capacity of Spd to muscle-type nAChR as well as α7 nAChR was more than five-fold lower than that of Spm, while Put was almost inactive. The replacement of the amino group with the guanidino group in some cases is known to enhance pharmacological activity of the compound. Thus, guanidine-containing analogs of amantadine (1-aminoadamantane) and memantine (3,5-dimethyl-1-aminoadamantane), i.e., 1-adamantylguanidine and 3,5-dimethyl-1-adamantylguanidine, have better therapeutic indices than memantine (Gmiro and Serdyuk, 2021). This is in line with the effects of Put and Agm on the muscle-type *T. californica* nAChR (Figure 3A). Moreover, the longest in chain guanidine-C5 (Gua-C5, Figure 1) was the most active among Agm analogs, although it failed to reach the effectiveness of Spm (Figure 3A). The binding capacity of the six diastereomers of *bis*-methylated Spm analogs (Figure 1) to muscle-type *T. californica* nAChR was almost identical, and similar to that of natural Spm (Figure 3C; Supplementary Figure S1; Table 1). The inhibition curves were plotted for two diastereomers of 1,12-Me_2_Spm and they were almost identical, but differed from that of Spm (Figure 3C), while their IC_50_ values were very similar (Table 1). Interestingly, the IC_50_ value for AgTx-636 (Figure 1), the central fragment of which is Spm-like 1,13-diamino-4,8-diazatridecane with *N* and *N*′-terminal substituents, was only two times lower compared to that of Spm (Table 1).

**TABLE 1:** Competition of Spd, Spm, diastereomers of 1,12-Me_2_Spm, and AgTx-636 with ^125^I-αBgt for binding to *T. californica* nAChR.

Compound	IC_50_ ± SEM (μM)
Spd	˃150
Spm	28.6 ± 3.7
(S,S)-1,12-Me_2_Spm	31.0 ± 3.9
(R,R)-1,12-Me_2_Spm	30.8 ± 4.3
AgTx-636	15.3 ± 1.3

Since we observed that Spm, Spd, and Agm can orthosterically bind to human α7 nAChR (competing with ^125^I-αBgt), we checked their capability to inhibit this receptor subtype. The obtained calcium imaging results showed only a tendency of inhibition (around 20%) of PNU282987-evoked response by Spm and Agm (Figure 2C). Electrophysiological studies showed a very weak inhibition of rat α9α10 nAChR expressed in *Xenopus* oocytes by *rac*-2,11-Me_2_Spm (IC_50_ ≈ 500 μM, and no inhibition was observed for Spm at 500 μM (Figure 2D).

### 3.3 Interaction of argiotoxin-636 with muscle-type, α7 and α9α10 nAChRs

We studied the inhibitory activity of AgTx-636 toward both muscle-type and neuronal nAChRs, paying special attention to its interaction with α9α10 subtype. In competitive radioligand analysis we observed inhibition of ^125^I-αBgt binding to muscle-type *T. californica* nAChR (IC_50_ ≈ 15 μM, Table 1). In contrast, its interaction with human α7 nAChR expressed in GH4C1 cells was very weak. At a concentration of 50 μM, AgTx-636 inhibited ≤20% of ^125^I-αBgt binding to this nAChR subtype (Figure 3D).

According to calcium imaging data, at a concentration of 10 μM, AgTx-636 was able to inhibit the action of a specific α7 nAChR agonist PNU282987 (200 nM) by ≥ 70% (Figure 2E), while α-cobratoxin (CTX) at a lower concentration of 5 μM completely suppressed the activity of this receptor (Figures 2C, E). It is difficult to accurately determine the percentage of the inhibitory effect of AgTx-636, since this toxin alone caused a noticeable [Ca^2+^]_i_ increase in human neuroblastoma SH-SY5Y cells [without α7 nAChR activation; Figure 2E, bar 4 (AgTx-636 “+”)]. This AgTx-636-evoked [Ca^2+^]_i_ increase remains visible in the presence of PNU282987 or CTX (Figure 2E, bars 3 and 5). Therefore, in addition to binding to α7 nAChR, AgTx-636 also acts on some other target in SH-SY5Y cells. Such combination of inhibitory and activating effects of AgTx-636 is apparently due to its ability to bind to various molecular targets in neuroblastoma cells. The high-affinity targets of AgTx-636 and other acylpolyamines are calcium-permeable glutamate receptors, which are blocked in the nanomolar range (Hansen et al., 2021). At higher concentrations AgTx-636 and other acylpolyamines affect a range of targets including different ion channels and receptors (Vassilevski et al., 2009). Unfortunately, at this time it is not possible to figure out which target is affected in SH-SY5Y cells. Our electrophysiological study showed an effective inhibition of rat α9α10 nAChR expressed in *Xenopus* oocytes by AgTx-636 (IC_50_ = 220 ± 22 nM, Figure 2F).

## 4 Discussion

Here, we tested the inhibitory activity of the disulfide-fixed octa-arginine “cyclo”R8, a series of cationic amino and guanidino group-containing compounds including biogenic polyamines such as Agm, Put, Spd, and Spm, *bis*-methylated Spm analogs, and spider venom-derived toxin AgTx-636 against muscle-type, α7 and α9α10 nAChRs. Our attention was mostly focused on the inhibition of α9α10 nAChR, since it opens the possibility to develop new effective drugs reducing neuropathic pain (Hone and McIntosh, 2023; Shelukhina et al., 2023). In this connection testing the interaction of the polyamine-related compounds with α7 nAChR seemed necessary because activation of this receptor subtype is important in the cholinergic anti-inflammatory pathway (Zhou et al., 2022; Shelukhina et al., 2023) and its inhibition would limit possible application of polyamines as probable analgesics. As can be seen from Figure 2A, “cyclo”R8 inhibits α9α10 nAChR almost as effectively as R8 itself (Lebedev et al., 2019). There are also no considerable differences between these two compounds in their inhibition of α7 nAChR (Figure 2B). Thus, a new “cyclic” octa-arginine peptide R8, the *N*- and *C*-termini of which are connected by the added cysteine residues with the closed disulfide, preserves the high affinity of the original linear R8 peptide to α9α10 and α7 nAChRs. The resulting activity of “cyclo”R8 can be the basis for creating new compounds sharing such structure with a desired biological activity.

Biogenic polyamines Spd and Spm are essential, ubiquitous organic polycations present in all eukaryotic cells in μM–mM concentrations and vitally important for the differentiation, proliferation and normal functioning of cells (Miller-Fleming et al., 2015; Pegg, 2016). Disturbances of polyamine metabolism are associated with many diseases (Nakanishi and Cleveland, 2021), including malignant tumors, since cancer cells have an elevated level of polyamines (Casero et al., 2018). It is well known that polyamine level is a marker for different neurodegenerative diseases (Seidl et al., 1996; Lewandowski et al., 2010; Inoue et al., 2013; Vrijsen et al., 2023) and corresponding enzymes of polyamine metabolism are potential therapeutic targets. An example of this is the successful application of the combination of DFMO (difluoromethyl ornithine, a specific irreversible inhibitor of ornithine decarboxylase, the rate-limiting enzyme of polyamine biosynthesis) and a polyamine uptake inhibitor AMXT 1501 for the treatment of neuroblastoma (https://www.ccia.org.au/our-impact/a-new-treatment-for-neuroblastoma). Simultaneous blocking of the synthesis and uptake of polyamines with DFMO and AMXT 1501 effectively starves the tumor cells of polyamines, significantly reducing the growth of diffuse intrinsic pontine glioma cells (Khan et al., 2021).

The interaction of *C*-methylated analogs of Spm with enzymes of Spm catabolism and cancer cells may be modulated by moving the methyl group along the Spm backbone (Khomutov et al., 2019b). The introduction of methyl group(s) into the backbone of polyamine molecule leads to the appearance of a chiral center. This opens the possibility for the regulation of the biochemical properties of polyamine analogs by changing the configuration of thus formed chiral center as it was observed for the enzymes of polyamine metabolism and different cancer cells (Hyvönen et al., 2007; Hyvönen et al., 2011; Hyvönen et al., 2015; Keinänen et al., 2018). Respectively, we assumed that the efficiency of the interaction of *C*-methylated analogs of Spm with nAChRs might depend on the position of methyl group and/or stereo configuration of chiral centers.

Here, we checked the binding of naturally occurring polyamines, *C*-methylated analogs of Spm, as well as of agmatine and its analogs to the muscle-type nAChR of *T. californica* and to neuronal human α7 nAChR (Figure 3A, Figure 3B), the activity of some being tested on human α7 and rat α9α10 nAChRs (Figures 2C, D). In all cases a comparatively weak affinity was found. The activities of Spm and *bis*-methylated Spm analogs, including diastereomers, were similar toward muscle-type and neuronal α7 nAChRs, and only on α9α10 nAChR *rac*-2,11-Me_2_Spm was more potent than Spm, but still remained a very weak inhibitor (Figure 2D, IC_50_ of about 500 µM). This finding is of importance, since some of the *C*-methylated Spm derivatives are considered as potential drug candidates, so due to their low affinity there is no need to expect their undesirable effects on nAChRs. Interestingly, for both natural polyamines and agmatine analogs the binding capacity correlated with their chain length: the most active were the compounds with the longest polyamine chain, namely, Spm and diastereomers of Me_2_Spm.

We found that AgTx-636 inhibited αBgt binding to the muscle-type *T. californica* nAChR (IC_50_ ≈ 15 μM, Figure 3D; Table 1), its affinity being close to that of Spm and diastereomers of 1,12-Me_2_Spm (IC_50_ ≈ 30 μM, Figure 3C; Table 1). The similar IC_50_ values may indicate that in this case the polyamine backbone is responsible for the binding efficiency. AgTx-636 showed a very low affinity to the orthosteric ligand-binding site of α7 nAChR, competing only for ≤20% of ^125^I-αBgt binding sites in GH4C1 cells at 50 μM (Figure 3D). On the contrary, the calcium imaging experiments demonstrated a potent inhibitory action of AgTx-636 on human α7 nAChR in neuroblastoma SH-SY5Y cells (Figure 2E). Probably, in this case AgTx-636 acts as a pore blocker, similarly to its mode of action on glutamate receptors (Twomey et al., 2018). AgTx-636 also produced a weak [Ca^2+^]_i_ increase independent of nAChR activation or inhibition, suggesting that there is an additional target for the toxin in these cells. The most important result is a comparatively high inhibitory activity of AgTx-636 against rat α9α10 nAChR (IC_50_ ≈ 200 nM) revealed by electrophysiological analysis (Figure 2F). Thus, in electrophysiological tests AgTx-636 has some advantages as compared to αBgt, which has a similarly high affinity to the muscle-type, α7 and α9α10 nAChRs.

In addition, it is well known that the receptors targeted by polyamine-related compounds, i.e., glutamate receptors and nAChRs, are widely expressed in glial cells. For example, astrocytes express α4* (Ma et al., 2023) and α7 nAChRs (Sharma and Vijayaraghavan, 2001; Fontana et al., 2023), which are involved in the pathogenesis of Alzheimer’s and other diseases. α9 subunit is expressed in glial type-II vestibular hair cells of the inner ear (Kong et al., 2006). Glial cells express functional AMPA and NMDA-type glutamate receptors (Burnashev et al., 1992; Lalo et al., 2006). We therefore anticipate that the compounds analyzed in this communication will be used to study not only neurons but also glia.

We conclude that oligoarginines and some low-molecular-mass polycationic compounds effectively inhibiting α9α10 nAChR may serve as a basis for the development of analgesics to reduce neuropathic pain.

## Data Availability

The raw data supporting the conclusions of this article will be made available by the authors, without undue reservation.

## Abbreviations

nAChR, nicotinic acetylcholine receptor; R8, octa-arginine; CTX, α-cobratoxin; Spm, spermine; Spd, spermidine; Agm, agmatine; Me_2_Spm, *C*-methylated spermine; AgTx-636, argiotoxin-636; AMPA, α-amino-3-hydroxy-5-methyl-4-isoxazolepropionate; NMDA, *N*-methyl-D-aspartate; ^125^I-αBgt, ^125^I-α-bungarotoxin.
