# Supplementary material for: Inhibition of nicotinic acetylcholine receptors by oligoarginine peptides and polyamine-related compounds
Source: Front Pharmacol. 2023 Dec 15;14:1327603. doi: 10.3389/fphar.2023.1327603 (PMC10758494; doi:10.3389/fphar.2023.1327603)
Supplement: Supplementary file 1 [file DataSheet1.docx]

**Supplementary materials**


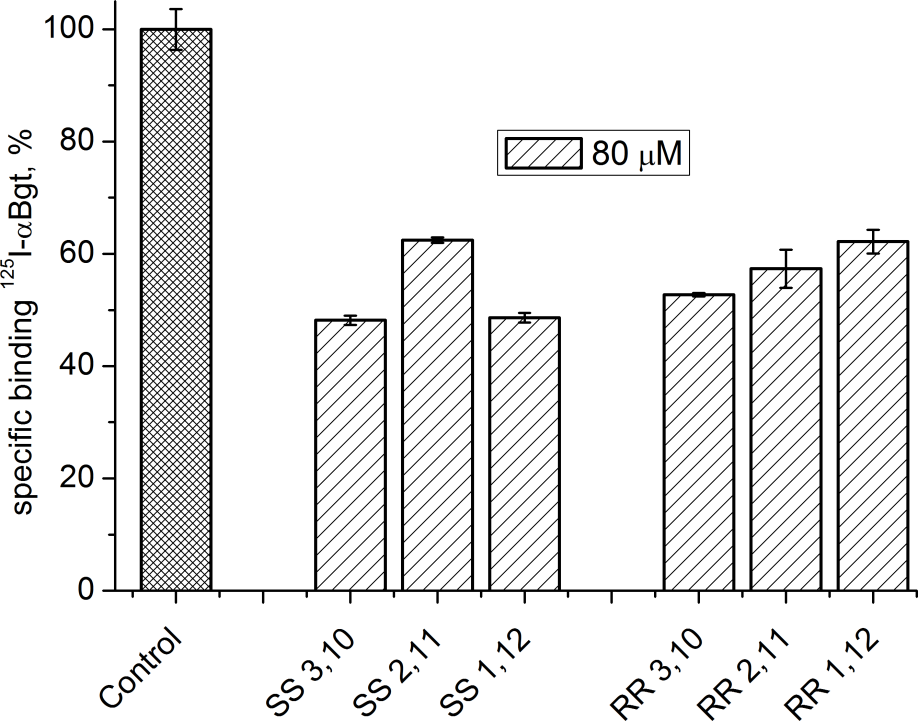


**Figure S1.** Inhibition of ^125^I-αBgt specific binding to muscle-type *T. californica* nAChR by six diastereomers of *bis*-methylated Spm analogs (80 μM). Specific binding of ^125^I-αBgt in the absence of compounds was accepted as 100%. Each point is represented as the mean ± SEM of 2–4 independent experiments.
